# Approaches to Facilitate Improved Recruitment, Development, and Retention of the Rural and Remote Medical Workforce: A Scoping Review Protocol

**DOI:** 10.34172/ijhpm.2020.27

**Published:** 2020-03-10

**Authors:** Farah Noya, Kirsty Freeman, Sandra Carr, Sandra Thompson, Rhonda Clifford, Denese Playford

**Affiliations:** ^1^Division of Health Professions Education, Faculty of Health and Medical Sciences, University of Western Australia, Perth, WA, Australia.; ^2^Western Australian Centre for Rural Health, The University of Western Australia, Perth, WA, Australia.; ^3^School of Allied Health, Faculty of Health and Medical Sciences, University of Western Australia, Perth, WA, Australia.; ^4^The Rural Clinical School of WA, School of Medicine, Faculty of Health and Medical Sciences, The University of Western Australia, Perth, WA, Australia.

**Keywords:** Recruitment and Retention, Medical Workforce, Rural and Remote, Workforce Development, Job Satisfaction

## Abstract

**Background:** Medical workforce scarcity in rural and remote communities is a global problem, severely challenging healthcare delivery and health equity. Both developed and developing countries report geographically uneven distributions of the medical workforce. This scoping review synthesizes evidence from peer-reviewed and grey literature concerning approaches implemented to improve the recruitment, development, and retention of the rural medical workforce in both developed and developing countries.

**Methods:** We will utilize the Arksey and O’Malley (2005) framework as the basis for this scoping review. The databases to be searched include Medline, Embase, Global Health, CINAHL Plus, and PubMed for articles from the last decade (2010-2019). Searches for unpublished studies and grey literature will be undertaken using the Google Scholar - Advanced Search tool. Quantitative and qualitative study designs will be included. Two authors will independently screen and extract relevant articles and information, with disagreements resolved by a third. Quantitative and qualitative analyses (thematic) will be conducted to evaluate and categorize the study findings.

**Discussion:** The scoping review will aid in mapping the available evidence for approaches implemented to advance the process of recruitment, development, and retention of the medical workforce in the rural and remote areas in developed and developing nations.

## Background

The World Health Organization (WHO)^1^ declared providing “health for all” to achieve the Sustainable Development Goals as their health priority. However, this aim is still far from being realized, with one out of every 17 of the world’s citizens – approximately 400 million people – lacking access to primary health services, predominantly in developing countries.^1^ Key to improving the health status of individuals is ensuring that communities gain access to health services, health facilities, and appropriate health professional care. However, medical workforce shortages and maldistribution of the workforce remain critical issues for many in rural and remote communities. Studies have shown that the problems of recruitment and retention of the medical workforce in rural and remote areas occur in most countries, whether developed or developing.^2-5^ Even the United States, Canada, and Australia with their advanced technologies in health and medicine such as telehealth have ongoing rural medical workforce shortages.^2-5^

Medical workforce shortages occur across both primary and specialist care, and what is counted and readily available workforce statistics differ within and by country. The USA, a vastly diverse country, has long documented a shortage of doctors in rural areas.^6^ The ratio of patients to primary care physicians in rural areas is only 39.8 physicians per 100 000 people, compared to 53.3 physicians per 100 000 in urban areas.^7^ Similarly, Australia experiencing an ongoing rural medical workforce shortage.^8^ In remote/very remote areas there are only 239 doctors per 100 000 people, compared to 403 doctors per 100 000 in urban areas.^9^ Canada is also experiencing a shortage of rural doctors, with 241 doctors per 100 000 people, lower in rural area than in urban area.^10^ What is important is that in all these countries, the workforce shortage occurs where residents have an overall higher burden of disease, and this is manifest in rural-urban disparities in health status,^8,11-16^ poorer access to healthcare^17-19^ and lower life expectancy.^20,21^

Despite similarities with developed countries in health-related challenges and disparities in urban-rural health status, a more complex health-related situation occurs in developing countries. India is the second most populous country in the world and has just achieved the WHO recommendation of a ratio of doctors to population of 1:1000 in 2017, although this counts both registered medical practitioners of modern medicine and traditional medicine.^22^ In India, the rate of medical doctors is 7.776 per 10 000 population with substantial differences in allopathic doctor distribution: the rate of allopathic doctors to population is around four times in urban (13.4/10 000) compared to rural areas (3.6/10 000) areas.^23^ The majority of India’s people, and most of its poor, still live in rural India. Communicable diseases outbreaks and epidemics are major health challenges,^24^ while non-communicable diseases contribute to 60% of deaths.^25^ Infant and maternal mortality rates are also high in rural India.^26^ The rural health challenges are complex, persistent and contribute to a worsening quality of life in rural India. Another developing country with documented problems and solutions to medical workforce shortage is the Philippines. As an archipelago of 7107 islands, there are difficulties ensuring adequate medical workforce and health services for rural and remote areas.^27^ However, the Philippines has documented an approach through socially accountable-community engaged medical education which has had a positive impact on solving their challenges.^27-30^ Indonesia is another developing country with rural-urban difference health-related problem and healthcare access due to medical workforce shortage.^31-33^ Indonesia has a rate of doctors per head of population which is well below the WHO recommendation, with only 0.377 per 10 000 population in 2017, less than half of which are generalist medical practitioners.^34^ Although there are few reports on how they manage the problem of workforce shortage, several policies on recruitment and deployment of health workers have been implemented.^35^ Among the policies, compulsory service, contracted staff and special assignment of strategic health workers such as doctors have partially addressed the severe shortage of health workers in remote areas.^35^ However, these successes seem to be short term, with long term solutions needed for the ongoing problems that exist.^34^

In relation to development, recruitment, and retention of the rural and remote workforce, studies have shown that medical schools’ social accountability (which includes contextual learning) plays an important role in facilitating a sustainable rural and remote workforce.^5,36-46^ Government policy and incentives also assist in developing workforce sustainability.^47,48^ Intrinsic motivation also plays an important role in recruitment and retention.^49-53^

Rourke^41^ identified four key areas regarding the social responsibility of medical schools that were required to develop a sustainable rural and remote workforce: recruitment of students from rural areas, relevant contextual learning, postgraduate training, and professional development.^41^ Evidence from numerous studies supports this perspective.^5,36,42,43^ Social accountability that has been implemented by medical schools includes, but is not limited to, recruiting students of rural origin^5,38,44^ and training with rural learning experience.^39,45,46^ These actions have been proved to increase the intentions of medical graduates to work rurally and aid the recruitment of rural workforce.^5,38,44^

One of the WHO recommendations for improved retention of the rural workforce is through regulatory interventions.^47^ The interventions that can be implemented by government include the creation of conducive conditions for health providers, rapid training in order to fulfill the workforce demand, optimizing the use of bonded service and allocation of educational grants to compulsory rural assignments.^47^

The medical workforce requires a support structure in order to effectively function in rural and remote settings.^47,48^ Multiple studies and reviews recommend that such support include mentoring in the workplace, adequate facilities, appropriate equipment for service and educational purposes, adequate telecommunications to enable the support of urban expertise, adequate relief with respect to on-call work, and professional career development opportunities in rural areas.^47,48^

Financial inducement is another essential factor in recruiting and retaining the medical workforce globally, particularly in developing countries.^47,48^ This stimulus includes fiscal bonuses, in-kind benefits (a free house or vehicle), and any other benefits that reduce opportunity costs related to working in rural areas. Examples of opportunity costs include lost income because of limited opportunities for private practice in rural areas, and supplementary housing costs from maintaining a residence in an urban area (for children’s education and a spouse’s job).^47,48^ Discrete choice experiments in several developing countries reveal that doctors prefer employment in a rural area in specific conditions that reflect their interests.^54-57^ Therefore, it can be said that the recruitment and retention of the rural and remote workforce are partly determined and facilitated by financial schemes, such as generous salary and incentives.

In addition to external factors, intrinsic motivation and self-determination are drivers to remain and productively serve in underserved areas, often more than financial or external drivers.^58^ Intrinsic motivation inducements come from within the individual and are known to contribute to job fulfillment.^58^ For example, the intrinsic pleasure resulting from self-sufficiency or meeting challenges at work, and the feeling of making a valuable contribution to the community’s healthcare create personal satisfaction in an individual’s work and performance. Prosocial motivation is another dimension of intrinsic motivation proposed by Neumann and Ritz^59^ and drives individuals to contribute to public service even in the absence of job satisfaction or rewards. Individuals will contribute to the public goods because of the desire to exert effort to benefit others.^59^ These intrinsic motivations provide reasons beyond extrinsic incentives to take part in the work and contribute directly to job satisfaction.^50,59^ Evidence^50,53,56^ shows that intrinsic motivation drives doctors to work and remain in rural posts. It means that the recruitment and retention of the rural and remote medical workforce are also influenced by intrinsic values, social norms, and trust.

A preliminary search of MEDLINE, the Cochrane Database of Systematic Reviews and the *JBI Database of Systematic Reviews and Implementation Reports* identified no existing systematic reviews on the topic of interest or protocols flagging that such a review was underway. While there are a large number of reviews existing in the subject area of rural workforce recruitment and retention, most do not focus on medical workforce or focus on developed countries such as the United States, Canada, and Australia which have similar medical education and healthcare systems.^60-62^ Several reviews in this area have focussed on educational aspects, including looking at the impact of curriculum or training with rural approach on medical students’ intention and doctors’ preferences to work rurally.^13,46,63-66^ Other than medical education, a number of reviews have explored strategies to address service provision to rural and remote areas.^67^ However, these are all from the developed world, with the conditions pertaining to the developing world vastly different. The factors associated with rural work, the incentives that are effective, and the aspirations of rural practitioners may differ entirely from the developed world. These factors should also be critically explored from developing countries’ point of view as part of efforts to achieve health equity.

The objective of this review is to synthesize international evidence from peer-reviewed and grey literature of approaches that have been implemented with the aim of improving the recruitment, development, and retention of the rural medical workforce in both developed and developing countries. This review will include broad approaches and facilitators which have been utilized, looking at a wider range of strategies, distilling critical success factors from the international literature. This review will be valuable for any country aiming at improving recruitment and retention of their rural and remote medical workforce.

## Methods

The proposed scoping review will be conducted in accordance with the Arksey and O’Malley framework for scoping reviews.^68^ This protocol follows the relevant aspects of the Preferred Reporting Items for Systematic Review and Meta-Analysis Protocols (PRISMA-P) guidelines^69^ to ensure rigour in reporting the methodology in the interim, while PRISMA extension for scoping review (PRISMA-ScR)^70^ will be used as a guide to ensure robustness in reporting the findings of scoping reviews.

### Stage 1. Identifying the Research Questions

The research question was developed as a broad framing of the population (ie, the medical workforce), the concept (recruitment, development, and retention of the workforce) and the context (rural and remote areas in developed and developing countries) to be explored and mapped to the objectives of the review. To meet the objectives, we ask the following questions:

1. What factors have been proved to be determinants of the rural and remote medical workforce recruitment, development, and retention?
2. What strategies/approaches have been implemented to improve the recruitment, development, and retention of the rural and remote medical workforce?
3. What is the evidence of the success of these approaches?
4. What are the similarities and differences between approaches implemented in developed countries and developing countries?
5. How feasible is it to apply identified successful strategies/models to other countries and contexts?

### Stage 2. Identifying Relevant Studies

#### Search Strategy

The search strategy will source both published and unpublished studies. An initial limited search of MEDLINE and CINAHL Plus was undertaken to identify relevant articles. The words in the titles and abstracts of relevant articles and their index terms were used to develop a full search strategy for each relevant database (Supplementary file 1). This search strategy, including keywords and index terms, will be adapted for each included information source. The reference lists of all studies selected for critical appraisal will be screened for additional studies. The search strategy relates to the population (the medical workforce), the concept (recruitment, development, and retention of the workforce) and the context (rural and remote areas in developed and developing countries).

#### Information Sources

The databases to be searched include Medline, Embase, Global Health, CINAHL Plus, and PubMed. These databases comprehensively capture relevant health literature. Preliminary searching found that the Informit Health Collection identified articles that were duplicates or were not useful for this review, so this database will not be included. The initial search query was developed for Medline (Ovid, including In-Process and Other Non-Indexed Citation) with the advantage of using the MeSH terms to index the citations and a shared platform with Embase for a quicker translation of search strategy. Sources of unpublished studies and grey literature will be searched using the Google Scholar - Advanced Search tool.

### Stage 3. Study Selection

Following the search, all identified citations will be collated and exported in EndNote or PubMed XML format or using the RIS text format. These formats will ease the transfer to systematic review management software Covidence.^71^ Covidence supports import and deduplication of citations, title and abstract and full-text screening, risk of bias assessment and data extraction, and the exporting of data directly into Excel.^71^ Titles and abstracts will then be assessed for relevancy. To be relevant for full-text review, the title and abstract will need to include a focus on medical workforce, recruitment, retention, or development of the workforce in a rural or remote setting, and describe the implemented approach or strategy.

This scoping review will consider both experimental and quasi-experimental study designs including randomized controlled trials, non-randomized controlled trials, before and after studies, and interrupted time-series studies. In addition, analytical observational studies, including prospective and retrospective cohort studies, case-control studies, and analytical cross-sectional studies, will be considered for inclusion. This review will also consider descriptive observational study designs including case series, case studies and descriptive cross-sectional studies for inclusion. Qualitative studies including action research will also be considered. In addition, any systematic review that meets the inclusion criteria will be considered. Studies published since 2010 will be included to provide up-to-date evidence from the last decade.

Relevant articles resulting from the title and abstract screening will then be selected for full-text review and assessed in detail independently by a pair of reviewers to determine the eligibility against the inclusion criteria. As applied in the title and abstract screening, the articles must focus on the medical workforce, be about recruitment, or retention, or development of the workforce, and describe the implemented approach or strategy. Thus inclusion criteria to guide the assessment of each article are that the article:

1. Focuses on the medical workforce in rural and/or remote settings.
2. Describes rural and/or remote areas as the actual workplace of the medical workforce, not only perceptions/intentions/interests/career choices without evidence of their rural practice.
3. Describes the recruitment or development, or retention of the medical workforce.
4. Describes/discusses factors that influence positive recruitment or retention of the workforce, or the approach/strategy to improve the recruitment or development or retention of the rural medical workforce.

The articles have to meet all 4 criteria to be included in the full-text review. Articles will be excluded before data extraction according to these criteria:

1. No clear evidence the strategies/approaches influenced recruitment, or development or retention of a medical workforce in a rural or remote setting.
2. Full text is not written in English (Table).

**Table:** Inclusion and Exclusion Criteria

**Inclusion**	**Exclusion**
Focuses on the medical workforce in rural and/or remote settings	Broader focus on healthcare professionals
Describes rural and/or remote areas as the actual workplace of the medical workforce	Describes perceptions/intentions/interests/career choices without evidence of their rural practice
Describes the recruitment or development, or retention of the medical workforce	No clear evidence the strategies/approaches influenced recruitment or development or retention of a medical workforce in a rural or remote setting
Describes/discusses factors that influence positive recruitment or retention of the workforce, or the approach/strategy to improve the recruitment or development or retention of the rural medical workforce	Discusses the negative result of a strategy/approach

The expected evidence or influence of the approaches include, but not limited to: (1) as the impact of the strategy implemented, the practice location is in rural or remote areas; (2) the continuity in rural and remote practice; (3) low turnover; (4) job satisfaction; and (5) ongoing development of clinical skills/professional career.

At the end of the full-text screening, using Covidence,^71^ all included full texts will be allocated to the next phase of data extraction. Reasons for exclusion of full-text studies will be recorded and reported in the findings. Any disagreements that arise between the reviewers at each stage of the study selection process will be resolved through discussion, or with a third reviewer/moderator (author SC).

The results of the search will be reported in full in the final review and presented in a PRISMA flow diagram (Figure).^69^

**Figure:**
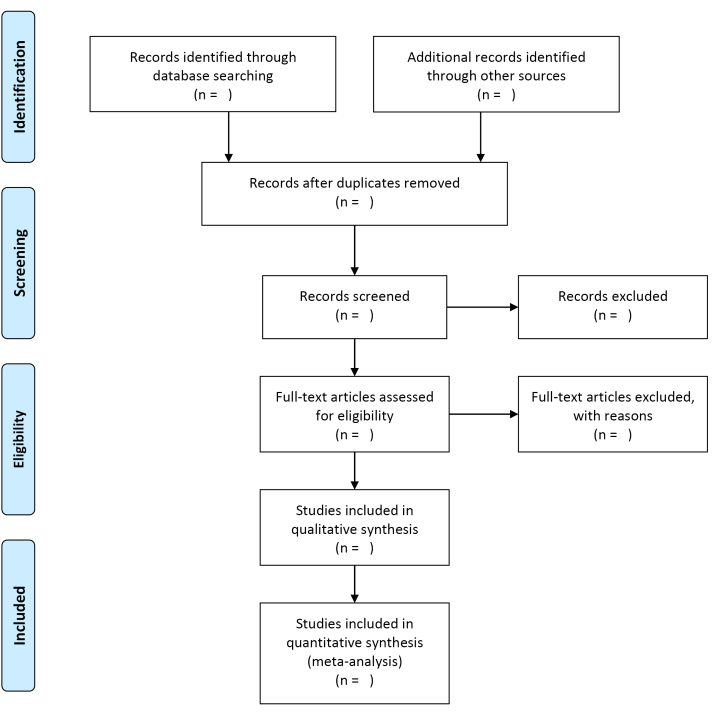
PRISMA^69^ Flowchart. Adapted from Moher D, Liberati A, Tetzlaff J, Altman DG. Preferred reporting items for systematic reviews and meta-analyses: the PRISMA statement. *Ann Intern Med.* 2009;151(4):264-269. doi:10.7326/0003-4819-151-4-200908180-00135. Abbreviation: PRISMA, Preferred Reporting Items for Systematic Reviews and Meta-Analyses.

### Stage 4. Charting the Data

#### Data Extraction

Data will be extracted from papers meeting the inclusion criteria by two independent reviewers using a data extraction tool customized by the reviewers from the data extraction tool template in Covidence.^40^ This data will include specific details about the population, concept, context, study methods, and key findings relevant to the review objective. A draft charting table is provided (Supplementary file 2). The data extraction tool will be modified and revised as necessary during the process of extracting data from included studies with modifications detailed in the final scoping review report. Any disagreements that arise between the reviewers will be resolved through discussion, or with a third reviewer (author SC). Authors of papers will be contacted to request missing or additional data where required. The quality of the articles will be assessed using the Mixed Method Appraisal Tool (MMAT) version 2018^72^ to apply an inclusive approach to the review, but not to exclude articles where the study quality is rated as low. The MMAT is a critical appraisal tool designed for systematic mixed study reviews.^72^ This makes it possible to assess the methodological quality of qualitative research, randomized controlled trials, non-randomized studies, quantitative descriptive studies, and mixed methods studies.^72^ Information on this quality assessment tool is provided (Supplementary file 3).

#### Data Presentation

The extracted data will be charted in tabular form in a manner that aligns with the objective of this scoping review. A narrative summary will accompany the tabulated and/or charted results and will describe how the results relate to the objective and question/s of the review.

### Stage 5. Collating, Summarising, and Reporting the Results

A descriptive qualitative analysis will be carried out on the included studies, and a thematic analysis^73,74^ undertaken for each research question. Basic numerical analysis of the extent, nature, and distribution of the studies included in the review will be explained using tables and charts. This will identify the dominant areas of research to facilitate improved recruitment and retention of the rural and remote medical workforce, as well as the research methods used and geographical distribution of the studies. The included studies will be organized thematically according to types of facilitation, theoretical, and conceptual positions adopted by authors. The themes will be reported to highlight the similarities, patterns, differences, and outliers found in the literature.

Besides PRISMA^69^ diagram for reporting of reviews, we will develop a final report of the review according to PRISMA-ScR guidelines.^70^

## Discussion

This study aims to synthesize positive evidence from programs or approaches implemented to address the problem of medical workforce shortage in developed and developing countries. Therefore, this scoping review will aid in mapping the available evidence for approaches implemented to advance the process of recruitment, development, and retention of the medical workforce in the rural and remote areas in developed and developing nations. This protocol outlines a rigorous study design using a recognized approach for scoping reviews, and a search strategy iteratively built in consultation with an experienced medical librarian. Quality assessment will be conducted in this review to provide a general picture of the quality of the studies included, although the approach is inclusive of all types of information available.

The results will be categorized under developed and developing countries according to the level of the approach being taken, whether they are at medical school, university, or government policy level. This categorization will assist stakeholders in identifying the most suitable approach within their level and condition.

The results of this review can inform stakeholders in needing countries of potential initiatives and policies used globally, that could aid recruitment, development and retention of medical workforce in the rural and remote areas. Adopting evidence-informed approaches can increase the probability of success in program implementation.

## Acknowledgments

The authors would like to acknowledge Ms. Terena Solomons, Medical Librarian (University of Western Australia, Perth, WA, Australia) who has provided invaluable expertise to the development and refinement of the search strategy of this scoping review.

## Ethical issues

This scoping study is a part of a project with several studies that have been granted Ethical Approval from The University of Western Australia No. RA/4/20/5065.

## Competing interests

Authors declare that they have no competing interests.

## Authors’ contributions

FN led the design and conceptualisation of this work, drafted the protocol, developed the search strategy, and conducted the search. KF, SC, ST, RC, and DP were involved in the conceptualisation of the review design, specifically in establishing the review question as well as the inclusion and exclusion criteria, provided feedback on the manuscript and copy-edited the manuscript. SC, ST, RC, and DP guided the conceptualisation and design of the study and data analyses and have revised all drafts of this manuscript for important intellectual content and clarity. All authors approve the publishing of this protocol.

## Authors’ affiliations

^1^Division of Health Professions Education, Faculty of Health and Medical Sciences, University of Western Australia, Perth, WA, Australia. ^2^Western Australian Centre for Rural Health, The University of Western Australia, Perth, WA, Australia. ^3^School of Allied Health, Faculty of Health and Medical Sciences, University of Western Australia, Perth, WA, Australia. ^4^The Rural Clinical School of WA, School of Medicine, Faculty of Health and Medical Sciences, The University of Western Australia, Perth, WA, Australia.

## Supplementary files

Supplementary file 1. Search Strategy.

Supplementary file 2. Charting Table.

Supplementary file 3. Mixed Methods Appraisal Tool Version 2018.
