# Supplementary material for: Approaches to Facilitate Improved Recruitment, Development, and Retention of the Rural and Remote Medical Workforce: A Scoping Review Protocol
Source: Int J Health Policy Manag. 2020 Mar 10;10(1):22–8. doi: 10.34172/ijhpm.2020.27 (PMC7947704; doi:10.34172/ijhpm.2020.27)
Supplement: Supplementary file 1 — Search Strategy. [file ijhpm-10-22-Supp1.pdf]

Supplementary file 1. Search Strategy

Database: Ovid MEDLINE(R) and Epub Ahead of Print, In-Process & Other Non-Indexed Citations, Daily and Versions(R) <1946 to March 15, 2019>

Search Strategy:

1. *Rural Population/
2. *Rural Health Services/
3. *Rural Health/
4. rural area*.ti,ab.
5. rural communit*.ti,ab.
6. rural location*.ti,ab.
7. rural practice*.ti,ab.
8. remote area*.ti,ab.
9. remote communit*.ti,ab.
10. remote location*.ti,ab.
11. remote practice*.ti,ab.
12. underserved area*.ti,ab.
13. underserved location*.ti,ab.
14. underserved communit*.ti,ab.
15. geographically isolated area*.ti,ab.
16. geographically isolated communit*.ti,ab.
17. island* communit*.ti,ab.
18. small island* communit*.ti,ab.
19. remote island* communit*.ti,ab.
20. poorly served area*.ti,ab.
21. poorly served communit*.ti,ab.
22. underserviced area*.ti,ab.
23. "rural and remote area*".ti,ab.

24. *Medically Underserved Area/

25. 1 or 2 or 3 or 4 or 5 or 6 or 7 or 8 or 9 or 10 or 11 or 12 or 13 or 14 or 15 or 16 or 17 or 18 or 19 or 20 or 21 or 22 or 23 or 24

26. *general practitioners/ or *physicians, family/ or *physicians, primary care/

27. *general practice/ or *family practice/

28. exp Medical Staff/

29. medical doctor.mp.

30. Medical officer*.ti,ab.

31. medical worker*.ti,ab.

32. medical profession*.ti,ab.

33. medical workforce.ti,ab.

34. medical graduate*.ti,ab.

35. health centre*.ti,ab.

36. medical centre*.ti,ab.

37. international medical graduate*.ti,ab.

38. foreign medical graduate*.ti,ab.

39. communit* medicine.ti,ab.

40. *Workforce/

41. *Physicians/sn, sd

42. 26 or 27 or 28 or 29 or 30 or 31 or 32 or 33 or 34 or 35 or 36 or 37 or 38 or 39 or 40 or 41

43. *personnel selection/ or *"personnel staffing and scheduling"/ or *personnel turnover/ or *staff development/ or *strikes, employee/ or *work engagement/ or *workplace/

44. *Job Satisfaction/

45. *Personnel Loyalty/

46. *Personal Satisfaction/

47. *Career Choice/

48. *Career Mobility/

49. personnel recruitment.ti,ab.

50. sustainable rural practice.ti,ab.

51. (sustain* adj3 employ*).ti,ab.

52. (attract* adj3 employ*).ti,ab.

53. personnel shortage*.ti,ab.

54. workforce shortage*.ti,ab.

55. "attract and retain".ti,ab.

56. "recruit and retain".ti,ab.

57. "recruitment and retention".ti,ab.

58. "recruiting and retaining".ti,ab.

59. (workforce adj3 maldistribut*).ti,ab.

60. under distribut*.ti,ab.

61. (commit* adj3 employ*).ti,ab.

62. *Motivation/ph, sn [Physiology, Statistics & Numerical Data]

63. (intrinsic adj3 motivat*).ti,ab.

64. (hire* adj3 staff).ti,ab.

65. improv* access.ti,ab.

66. engag* employ*.ti,ab.

67. interest* employ*.ti,ab.

68. (attract* adj3 employ*).ti,ab.

69. encourage* employ*.ti,ab.

70. work* satisfaction*.ti,ab.

71. (career adj3 advance*).ti,ab.

72. Unmet Need*.ti,ab.

73. workforce need*.ti,ab.

74. recruitment strateg*.ti,ab.

75. (retention adj2 strateg*).ti,ab.

76. career development.ti,ab.

77. (plan* adj5 workforce).ti,ab.

78. 43 or 44 or 45 or 46 or 47 or 48 or 49 or 50 or 51 or 52 or 53 or 54 or 55 or 56 or 57 or 58 or 59 or 60 or 61 or 62 or 63 or 64 or 65 or 66 or 67 or 68 or 69 or 70 or 71 or 72 or 73 or 74 or 75 or 76 or 77

79. *health plan implementation/ or *health priorities/

80. *Health Policy/

81. government initiative*.ti,ab.

82. support structure.ti,ab.

83. practical model*.ti,ab.

84. (rural adj3 package*).ti,ab.

85. alternative model*.ti,ab.

86. locum service*.ti,ab.

87. locum support.ti,ab.

88. (compulsory adj3 assignment*).ti,ab.

89. compulsory service*.ti,ab.

90. bond* scheme*.ti,ab.

91. bond* service*.ti,ab.

92. vacancy rate*.ti,ab.

93. utilization of service*.ti,ab.

94. duration of service*.ti,ab.

95. *Program Evaluation/

96. *Survival Analysis/

97. *regression analysis/

98. factor* impact*.ti,ab.

99. polic* analysis.ti,ab.

100. polic* initiativ*.ti,ab.

101. *physician incentive plans/ or *"salaries and fringe benefits"/

102. *Remuneration/

103. financial incentive*.ti,ab.

104. financial inducement*.ti,ab.

105. monetary incentive*.ti,ab.

106. (non-financial adj3 inducement*).ti,ab.

107. non-monetary incentive*.ti,ab.

108. incentiv* measure*.ti,ab.

109. incentiv* polic*.ti,ab.

110. *Socioeconomic factors/

111. exp Education, Medical/

112. faculty development.ti,ab.

113. professional development.ti,ab.

114. rural exposure.ti,ab.

115. rural learning experiences.ti,ab.

116. rural scholarship.ti,ab.

117. *Training Support/

118. educational grant*.ti,ab.

119. *Schools, Medical/

120. community participation.ti,ab.

121. social accountabilit*.ti,ab.

122. *Social Responsibility/

123. *Community-Institutional Relations/

124. 79 or 80 or 81 or 82 or 83 or 84 or 85 or 86 or 87 or 88 or 89 or 90 or 91 or 92 or 93 or 94 or 95 or 96 or 97 or 98 or 99 or 100 or 101 or 102 or 103 or 104 or 105 or 106 or 107 or 108 or 109 or 110 or 111 or 112 or 113 or 114 or 115 or 116 or 117 or 118 or 119 or 120 or 121 or 122 or 123

125. 25 and 42 and 78 and 124

126. limit 125 to (yr="2010 - 2019" and last 10 years)
