# Supplementary material for: Approaches to Facilitate Improved Recruitment, Development, and Retention of the Rural and Remote Medical Workforce: A Scoping Review Protocol
Source: Int J Health Policy Manag. 2020 Mar 10;10(1):22–8. doi: 10.34172/ijhpm.2020.27 (PMC7947704; doi:10.34172/ijhpm.2020.27)
Supplement: Supplementary file 2 — Charting Table. [file ijhpm-10-22-Supp2.pdf]

Supplementary file 2. Charting table

	**Description**
**Article details**	
Article type	
Year of publication	
Country	
Language	
Definition of rural/remote	
**Study details**	
Study design	
Participants	
Instrument/tools	
Intervention	
Comparator/control group	
Study outcomes	
Outcomes	
Power/significance of the study findings	
**Details of the program**	
Name	
Setting	
Participants	
Program delivery	
Facilitators	

Duration of the program/strategy	
Evaluation mechanism	
Timing of evaluation	
**Implementation factors**	
Success	
Evidence of effectiveness	
Enablers	
Recommendation	
